# Supplementary material for: Decrease in RNase HII and Accumulation of lncRNAs/DNA Hybrids: A Causal Implication in Psoriasis?
Source: Biomolecules. 2022 Feb 25;12(3):368. doi: 10.3390/biom12030368 (PMC8945458; doi:10.3390/biom12030368)
Supplement: Supplementary file 1 [file biomolecules-12-00368-s001.zip › biomolecules-1592890-supplementary.pptx]

## Slide 1
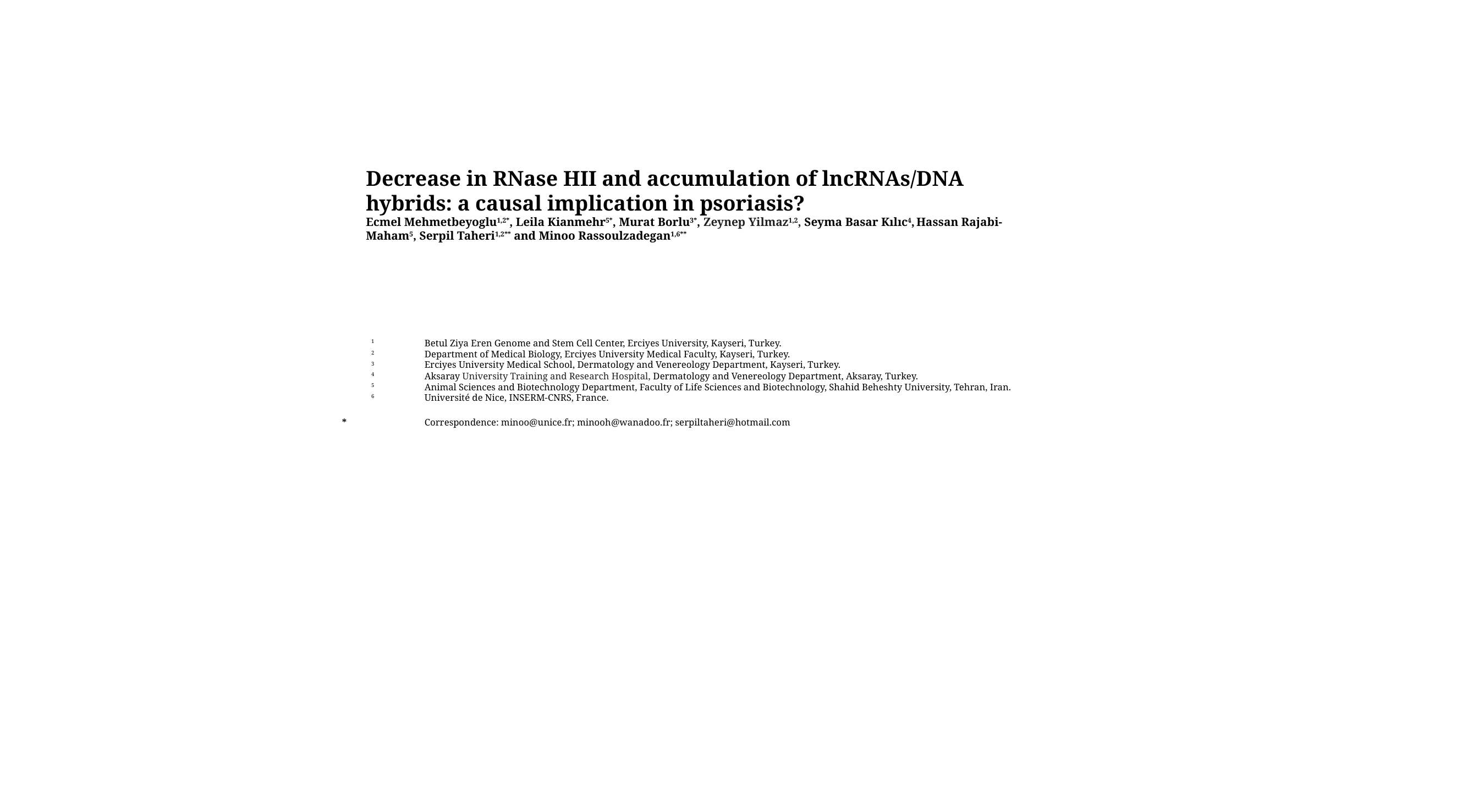

Decrease in RNase HII and accumulation of lncRNAs/DNA hybrids: a causal implication in psoriasis?
Ecmel Mehmetbeyoglu1,2*, Leila Kianmehr5*, Murat Borlu3*, Zeynep Yilmaz1,2, Seyma Basar Kılıc4, Hassan Rajabi-Maham5, Serpil Taheri1,2** and Minoo Rassoulzadegan1,6**
1	Betul Ziya Eren Genome and Stem Cell Center, Erciyes University, Kayseri, Turkey.
2	Department of Medical Biology, Erciyes University Medical Faculty, Kayseri, Turkey.
3 	Erciyes University Medical School, Dermatology and Venereology Department, Kayseri, Turkey.
4	Aksaray University Training and Research Hospital, Dermatology and Venereology Department, Aksaray, Turkey.
5	Animal Sciences and Biotechnology Department, Faculty of Life Sciences and Biotechnology, Shahid Beheshty University, Tehran, Iran.
6	Université de Nice, INSERM-CNRS, France.
*	Correspondence: minoo@unice.fr; minooh@wanadoo.fr; serpiltaheri@hotmail.com

## Slide 2
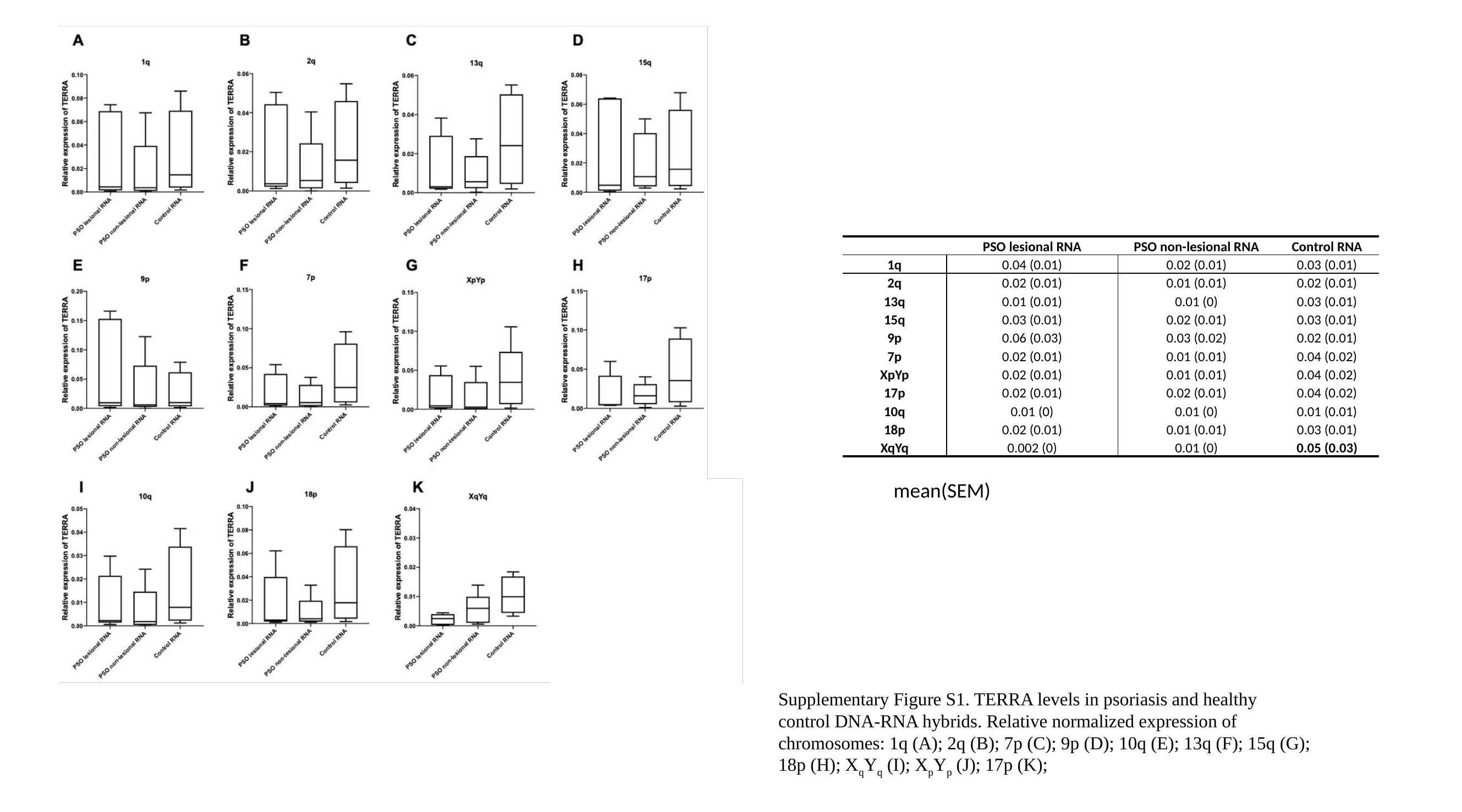

| | PSO lesional RNA | PSO non-lesional RNA | Control RNA |
| --- | --- | --- | --- |
| 1q | 0.04 (0.01) | 0.02 (0.01) | 0.03 (0.01) |
| 2q | 0.02 (0.01) | 0.01 (0.01) | 0.02 (0.01) |
| 13q | 0.01 (0.01) | 0.01 (0) | 0.03 (0.01) |
| 15q | 0.03 (0.01) | 0.02 (0.01) | 0.03 (0.01) |
| 9p | 0.06 (0.03) | 0.03 (0.02) | 0.02 (0.01) |
| 7p | 0.02 (0.01) | 0.01 (0.01) | 0.04 (0.02) |
| XpYp | 0.02 (0.01) | 0.01 (0.01) | 0.04 (0.02) |
| 17p | 0.02 (0.01) | 0.02 (0.01) | 0.04 (0.02) |
| 10q | 0.01 (0) | 0.01 (0) | 0.01 (0.01) |
| 18p | 0.02 (0.01) | 0.01 (0.01) | 0.03 (0.01) |
| XqYq | 0.002 (0) | 0.01 (0) | 0.05 (0.03) |
mean(SEM)
Supplementary Figure S1. TERRA levels in psoriasis and healthy control DNA-RNA hybrids. Relative normalized expression of chromosomes: 1q (A); 2q (B); 7p (C); 9p (D); 10q (E); 13q (F); 15q (G); 18p (H); XqYq (I); XpYp (J); 17p (K);

## Slide 3
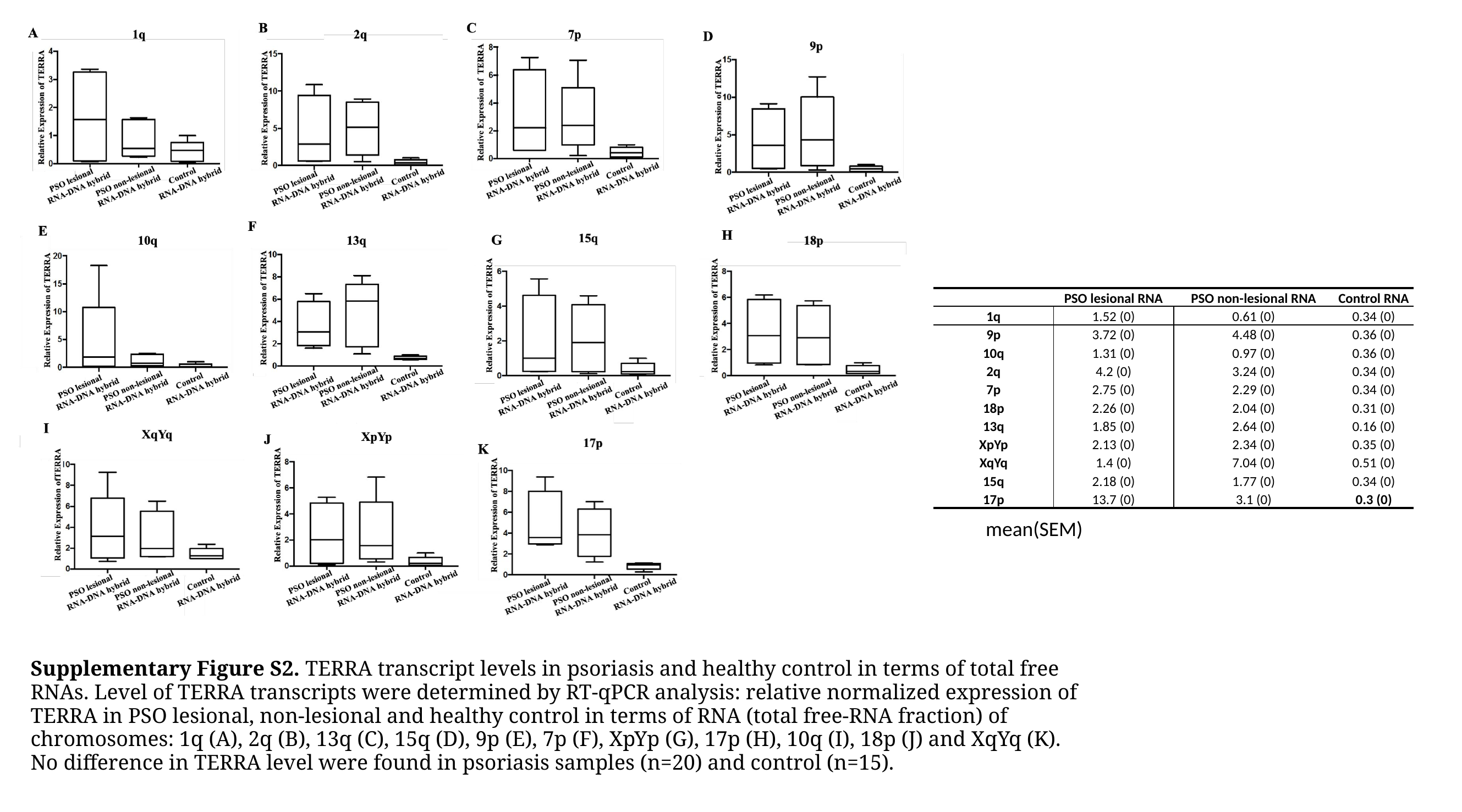

| | PSO lesional RNA | PSO non-lesional RNA | Control RNA |
| --- | --- | --- | --- |
| 1q | 1.52 (0) | 0.61 (0) | 0.34 (0) |
| 9p | 3.72 (0) | 4.48 (0) | 0.36 (0) |
| 10q | 1.31 (0) | 0.97 (0) | 0.36 (0) |
| 2q | 4.2 (0) | 3.24 (0) | 0.34 (0) |
| 7p | 2.75 (0) | 2.29 (0) | 0.34 (0) |
| 18p | 2.26 (0) | 2.04 (0) | 0.31 (0) |
| 13q | 1.85 (0) | 2.64 (0) | 0.16 (0) |
| XpYp | 2.13 (0) | 2.34 (0) | 0.35 (0) |
| XqYq | 1.4 (0) | 7.04 (0) | 0.51 (0) |
| 15q | 2.18 (0) | 1.77 (0) | 0.34 (0) |
| 17p | 13.7 (0) | 3.1 (0) | 0.3 (0) |
mean(SEM)
Supplementary Figure S2. TERRA transcript levels in psoriasis and healthy control in terms of total free RNAs. Level of TERRA transcripts were determined by RT-qPCR analysis: relative normalized expression of TERRA in PSO lesional, non-lesional and healthy control in terms of RNA (total free-RNA fraction) of chromosomes: 1q (A), 2q (B), 13q (C), 15q (D), 9p (E), 7p (F), XpYp (G), 17p (H), 10q (I), 18p (J) and XqYq (K). No difference in TERRA level were found in psoriasis samples (n=20) and control (n=15).
